# Characterization of the Soybean *GmCCS-GmCSN5B-GmVTC1* Pathway and Its Functional Roles Under *Soybean mosaic virus* Infection

**DOI:** 10.3390/plants15071020

**Published:** 2026-03-26

**Authors:** Bowen Li, Tao Wang, Mengzhuo Liu, Liqun Wang, Hui Liu, Tongtong Jin, Ting Hu, Kai Li, Haijian Zhi

**Affiliations:** 1 National Center for Soybean Improvement, National Key Laboratory for Crop Genetics and Germplasm Enhancement, Key Laboratory of Biology and Genetic Improvement of Soybean-Ministry of Agriculture, Nanjing Agricultural University, Nanjing 210095, China; 18838917013@163.com (B.L.); wt414210391@163.com (T.W.); 18851778923@163.com (M.L.); wanglq1124@126.com (L.W.); 2 Crop Research Institute, Hunan Academy of Agricultural Sciences, Changsha 410125, China; 3 Handan Academy of Agricultural Sciences, Handan 056001, China; 4 State Key Laboratory of Crop Gene Resources and Breeding, The National Key Facility for Crop Gene Resources and Genetic Improvement (NFCRI), Key Laboratory of Grain Crop Genetic Resources Evaluation and Utilization (MARA), Institute of Crop Sciences, Chinese Academy of Agricultural Sciences, Beijing 100081, China; qinghuan313718@163.com; 5 Xuzhou Institute of Agricultural Sciences of Xu-Huai Region of Jiangsu, Xuzhou 221131, China; shona126@sina.com; 6 Hunan Provincial Institute of Edible Fungus, Changsha, 410013, China; huting911025@163.com

**Keywords:** soybean, *Soybean mosaic virus*, *GmCSN5B*, *GmVTC1*, ascorbic acid (AsA)

## Abstract

*Soybean mosaic virus* (SMV) is a major constraint on global soybean (*Glycine max* (L.) Merr.) production, causing substantial economic losses worldwide. Despite these losses, the potential of resistance genes as a solution remains largely unexplored. In this study, the COPPER CHAPERONE FOR SUPEROXIDE DISMUTASE (GmCCS) was initially employed as a bait to screen the soybean cDNA library, leading to the identification of a protein homologous to *Arabidopsis thaliana* COP9 signalosome complex subunit 5B (AtCSN5B), designated as GmCSN5B. Quantitative real-time PCR (qRT-PCR) analysis revealed differential expression of *GmCSN5B* in the SMV-resistant (Qihuang No.1, QH) and susceptible (Nannong 1138-2, NN) variety following SMV-SC3 strain inoculation. Knockdown of *GmCSN5B* via *Bean pod mottle virus* (BPMV)-induced gene silencing (VIGS) significantly enhanced SMV resistance compared to control plants. This work further demonstrated that GmCSN5B can interact with the downstream GmVTC1 protein, which was potentially associated with ascorbic acid (AsA; Vitamin C) synthesis. Moreover, *GmVTC1* also responded to SMV infection, and its knockdown led to a reduction in endogenous AsA levels within the host, thereby compromising the plant’s resistance to SMV. Together, these findings suggest that the *GmCCS-GmCSN5B-GmVTC1* pathway in soybean modulates host resistance to SMV through the regulation of AsA synthesis.

## 1. Introduction

As the primary global source of edible vegetable oil and protein, soybean is continually threatened by numerous disease-causing organisms during its growth cycle. Among these pathogens, *Soybean mosaic virus* (SMV) is a single-stranded positive-sense RNA virus with a genome approximately 9.6 kb in length, which encodes 11 functionally distinct proteins [1,2]. SMV primarily invades host plants via mechanical damage, insect-mediated transmission and seed transmission [1,3]. Multiple dominant resistance loci, such as the *Rsv1*, *Rsv3*, *Rsv4*, *Rsc3*, *Rsc4*, have been genetically mapped or isolated. Most of these loci encode typical NBS-LRR type immune receptors that confer gene-for-gene resistance to specific SMV strains [4,5,6,7,8]. Distributed across major soybean-producing regions globally, including China, the United States, Brazil, and Argentina, SMV causes substantial damage on soybean yield and quality [2]. To date, no effective and environmentally sustainable chemical controls are available, making resistance gene mining and the breeding of resistant varieties the most practical and long-term management strategies.

Plant physiological processes and stress responses are intricately regulated by protein–protein interactions. Identifying proteins that interact with key immune or metabolic components thus provides valuable insights into plant–environment and plant–pathogen interactions [9,10]. For instance, screening a soybean cDNA library using SMV-6K1 as bait identified 127 potential interactors, and transient overexpression of *GmPR4* and *GmBI1* reduced SMV accumulation in *N. benthamiana* [11]. Similarly, the 14-3-3h protein interacted with the translationally controlled tumor protein (TCTP) of *Potato virus Y* (PVY), and its overexpression suppressed PVY replication [12]. Prior research demonstrated that *GmCCS* plays a crucial role in soybean defense against SMV infection [13]. This current study further investigated the function of GmCCS-interacting protein to elucidate the underlying resistance mechanism.

Ascorbic acid (AsA) is a water-soluble antioxidant that effectively scavenges cellular reactive oxygen species (ROS) and maintains redox homeostasis [14,15]. Moreover, it is pivotal for plant growth, development and stress response [16,17]. In plant–virus interactions, AsA modulates ROS bursts, defense signaling, hypersensitive cell death and viral replication and movement. Manipulation of AsA levels or biosynthesis has been shown to alter host susceptibility to multiple viruses including TMV, PVY and CMV [18,19]. Several AsA biosynthesis pathways have been identified [20,21,22], among which the _D_-Man/_L_-Gal pathway is predominant. GDP-Mannose pyrophosphorylase (GMPase, also known as VTC1) acts as a key enzyme in this pathway, catalyzing the conversion of _D_-Mannose-1-P to GDP-_D_-Mannose [23].

The CSN is a highly conserved multi-protein complex in the ubiquitin–proteasome pathway, first identified in *Arabidopsis thaliana* [24]. Among its eight subunits, CSN5 is distinct due to its dual function as both the catalytic center of the CSN complex and an independent regulator [24]. *AtCSN5* comprises two coding paralogs, *AtCSN5A* and *AtCSN5B*, exhibiting 94% protein sequence similarity. Nevertheless, the functions of CSN5B remain poorly characterized [25,26]. AtCSN5B interacted with AtVTC1 to control its degradation, influencing the VC levels and oxidative stress responses [27]. Similarly, tomato zinc finger protein (SlZF3) could directly bind to CSN5B and inhibit its interaction with VTC1, promoting the AsA accumulation and salt tolerance [28]. In soybean, *GmCSN5A* and *GmCSN5B* were induced under phosphate deficiency and modulated anthocyanin biosynthesis [29]. However, the biological function of the *CSN5B-VTC1* module in soybean under biotic stress, especially during SMV infection, remains completely unknown.

In this study, yeast two-hybrid library screening initially identified an interaction between GmCCS and GmCSN5B. Further analysis revealed that GmCSN5B also interacts with GmVTC1. Upon SMV induction, both *GmCSN5B* and *GmVTC1* exhibited differential expression patterns in resistant and susceptible soybean varieties. VIGS-based functional analysis demonstrated that *GmCSN5B* may negatively regulate the soybean resistance to SMV, while *GmVTC1* positively modulated host defense responses. Additionally, *GmVTC1* silencing obviously reduced endogenous AsA levels in soybean. Together, these findings reveal a novel *GmCCS-GmCSN5B-GmVTC1* module that confers SMV resistance, providing a theoretical basis for molecular breeding of SMV-resistant soybean varieties.

## 2. Results

### 2.1. Screening and Verification of Soybean Proteins Interacting with GmCCS

#### 2.1.1. Annotation of Proteins Interacting with GmCCS

**Figure 1:**
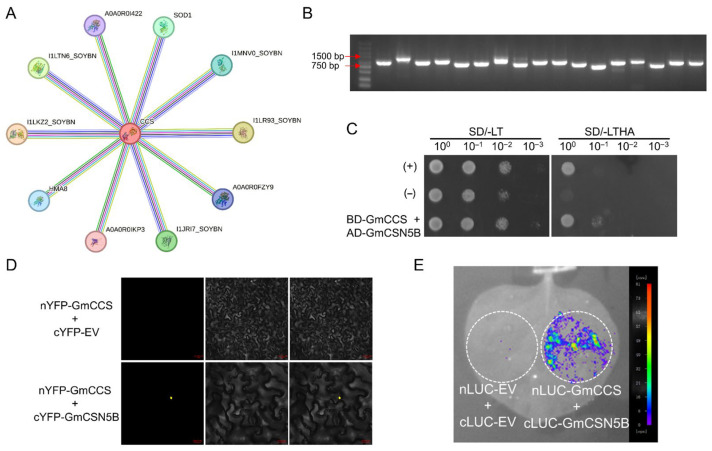
Screening of GmCCS-interacting proteins and validation of the interaction with GmCSN5B. (**A**) Using the STRING database, ten soybean proteins were predicted to potentially interact with GmCCS: I1LKZ2 (Glyma.11G192700), I1LTN6 (Glyma.12G178800), A0A0R0I422 (Glyma.09G052000), I1MNV0 (Glyma.16G153900), I1LR93 (Glyma.12G081300), A0A0R0FZY9 (Glyma.16G088300), I1JRI7 (Glyma.03G242900), A0A0R0IKP3 (Glyma.09G218700), Q7M1R5 (Glyma.19G240400) and I1K8G7(Glyma.06G056300). (**B**) Agarose gel electrophoresis analysis of PCR products derived from the extracted yeast plasmids. (**C**–**E**) The interaction between GmCCS and GmCSN5B was validated using Y2H (**C**), BiFC (**D**) and LUC (**E**) assays, respectively.

Bioinformatic predictions identified GmCCS as a central hub potentially interacting with 10 soybean proteins (Figure 1A; Table S1). To validate these interactions, we screened the soybean cDNA library using pGBK-GmCCS bait vector. PCR analysis performed on the extracted yeast plasmids identified the target bands, which ranged in size from 0.75 to 1.5 kb (Figure 1B). Sequencing and BLAST alignment (NCBI BLAST+ v2.15.0) against the soybean genome (*Glycine max* Wm82.a2.v1), followed by removal of redundant and non-matching sequences, yielded 11 candidate interactors. Gene annotation revealed that these proteins mainly included PEPTIDYL-PROLYL CIS-TRANS ISOMERASE CYP19-1 (Glyma.11G098700), DYNEIN LIGHT CHAIN (Glyma.17G095200), and others (Table 1). Interestingly, none overlapped with the originally predicted 10 proteins.

#### 2.1.2. Validation of the Interaction Between GmCCS and GmCSN5B

Among these proteins (Table 1, above), Glyma.06G076000 (GmCSN5B) was identified as a homolog of AtCSN5B (AT1G71230), which was a known stress-responsive protein [28,29]. Given this conserved function and homology, we selected GmCSN5B for further validation. Protein–protein interactions between GmCCS and GmCSN5B were consistently confirmed through the yeast two-hybrid (Y2H), bimolecular fluorescence complementation (BiFC) and dual-luciferase (LUC) assays (Figure 1C–E). Notably, the BiFC assay revealed nuclear-localized yellow fluorescence, demonstrating a specific interaction in the nucleus.

### 2.2. Characterization and Expression Analysis of GmCSN5B

#### 2.2.1. Sequence and Phylogenetic Analysis of GmCSN5B

**Figure 2:**
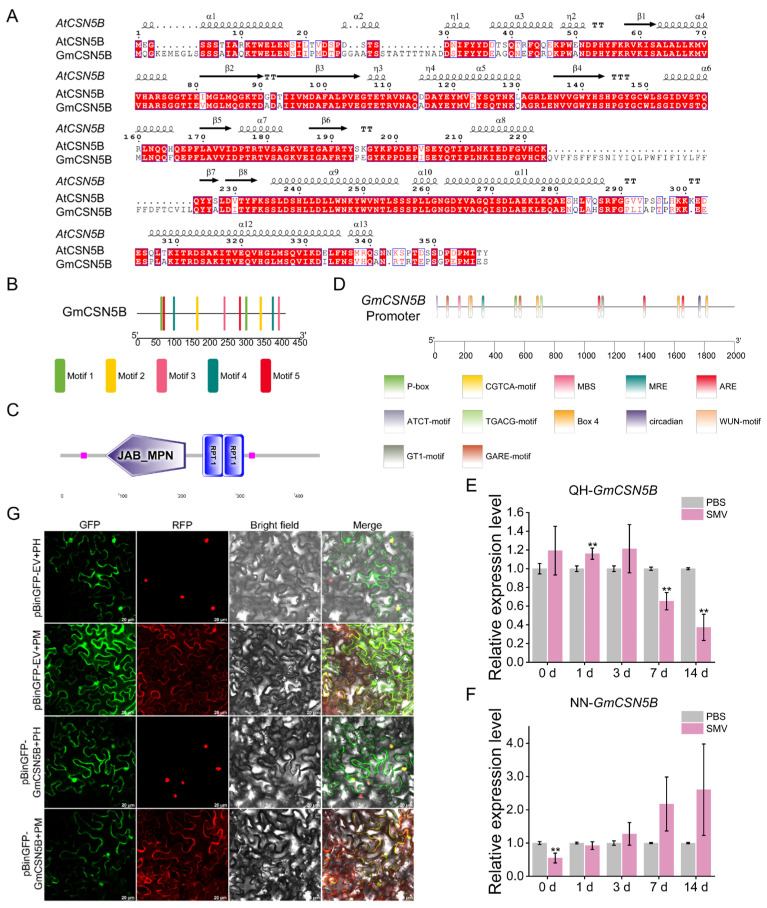
Sequence analysis, subcellular localization, and expression profiling of *GmCSN5B*. (**A**) Sequence alignment of GmCSN5B and AtCSN5B. (**B**,**C**) The conserved motifs (**B**) and domains (**C**) predicted for GmCSN5B. (**D**) Prediction of *cis*-acting elements in the upstream of *GmCSN5B* that may be related to stress response. (**E**,**F**) The expression levels of *GmCSN5B* in resistant variety QH (**E**) and susceptible variety NN (**F**) upon SC3 strain induction. The plants inoculated with PBS served as the control group. The *tubulin* was used as the internal reference gene. Values represented the means ± SD of three biological replicates. Differences were analyzed using One-Way ANOVA, **, *p* < 0.01. (**G**) Subcellular localization of GmCSN5B observed under the laser confocal microscope. Green fluorescence indicated GFP or GFP-fused protein, while red fluorescence represented membrane marker (PM) or nucleus marker (PH). Bar = 20 μm.

Sequence alignment using ESPript 3.0 showed a high degree of conservation between GmCSN5B and AtCSN5B (Figure 2A). Conserved domain analysis using SMART 9.0 identified a JAB-MPN domain (77–214 aa) at the N-terminus of GmCSN5B, along with two internal repeat domains (243–313 aa) in the central region (Figure 2B,C). Phylogenetic analysis using MEGA 7.0 revealed that GmCSN5B clustered most closely with its homolog from *Vigna unguiculata* (Figure S1A).

#### 2.2.2. The Expression Level Analysis of *GmCSN5B*

Tissue expression analysis revealed obviously higher *GmCSN5B* transcript levels in stems and leaves compared to other tissues (Figure S1B). Upon SMV infection, *GmCSN5B* exhibited contrasting expression patterns in QH and NN plants relative to their respective controls. In QH, expression was significantly upregulated at 1 day post-inoculation (dpi) before declining, while NN plants showed transient downregulation exclusively at 0 dpi (Figure 2E,F).

#### 2.2.3. Subcellular Localization of GmCSN5B

The prediction suggested that GmCSN5B may localize to multiple compartments, including the plasma membrane and endoplasmic reticulum. Experimental validation through *Agrobacterium*-mediated transient expression in *N. benthamiana* revealed predominant localization to the plasma membrane and nucleus (Figure 2G). This nuclear co-localization with GmCCS provides supporting evidence for their physical interaction.

### 2.3. Silencing of GmCSN5B Is Beneficial to Improve the Soybean Resistance to SMV

**Figure 3:**
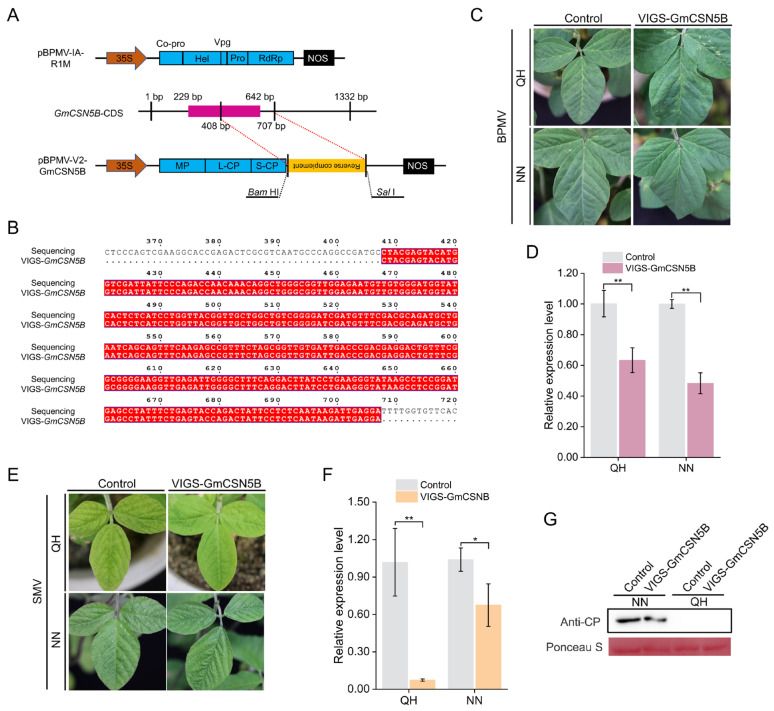
Functional validation of *GmCSN5B* using VIGS. (**A**) Schematic illustration of the pBPMV-V2-GmCSN5B vector construction strategy. (**B**) Sequencing analysis of the specific silencing fragment in the recombinant vector. (**C**) Leaf disease phenotypes of VIGS-GmCSN5B and control plants (inoculated with the pBPMV-V2 empty vector) at 7 days after the initial infection of BPMV. (**D**) qRT-PCR analysis of *GmCSN5B* silencing efficiency in QH and NN plants at 7 dpi. (**E**) Upper leaf phenotypes of the above plants at 10 dpi after SMV re-inoculation. (**F**) qRT-PCR analysis of *CP* gene expression levels in QH and NN plants, with *tubulin* as the internal reference gene. Values represented the means ± SD of three biological replicates. Differences were analyzed using Student’s *t*-test, *, *p* < 0.05; **, *p* < 0.01. (**G**) Western blot analysis of CP accumulation in the upper leaves after SMV re-inoculation, using Ponceau S staining Rubisco subunit as the internal reference protein.

A 300 bp fragment targeting *GmCSN5B* was designed and cloned into the pBPMV-V2 vector for VIGS assays (Figure 3A,B). Systemic viral infection was confirmed at 7 dpi based on typical yellow-green mottling symptoms (Figure 3C). Consistent with the phenotypic observations, qRT-PCR revealed significantly reduced transcript levels of *GmCSN5B* in VIGS-GmCSN5B plants from both QH and NN, relative to the corresponding control groups (Figure 3D).

The SC3 strain was subsequently inoculated onto the first trifoliolate leaves of both varieties. At 10 dpi, VIGS-GmCSN5B plants of QH and NN exhibited attenuated mosaic symptoms in the upper leaves compared to the control plants (Figure 3E). Consistent with these phenotypes, qRT-PCR confirmed significantly reduced transcript levels of the *CP* gene in the treated groups (Figure 3F). Western blot analysis further revealed decreased CP accumulation in NN, whereas no detectable CP was observed in QH (Figure 3G), suggesting the involvement of additional defense mechanisms in the resistant genotype. Combined with the induced expression pattern of *GmCSN5B* described in Section 2.2.2, these results indicated that *GmCSN5B* may negatively regulate soybean resistance to SMV.

### 2.4. Selection, Characterization and Expression Analysis of GmVTC1

#### 2.4.1. Sequence Analysis of All Candidate *GmVTC1* in Soybean

**Figure 4:**
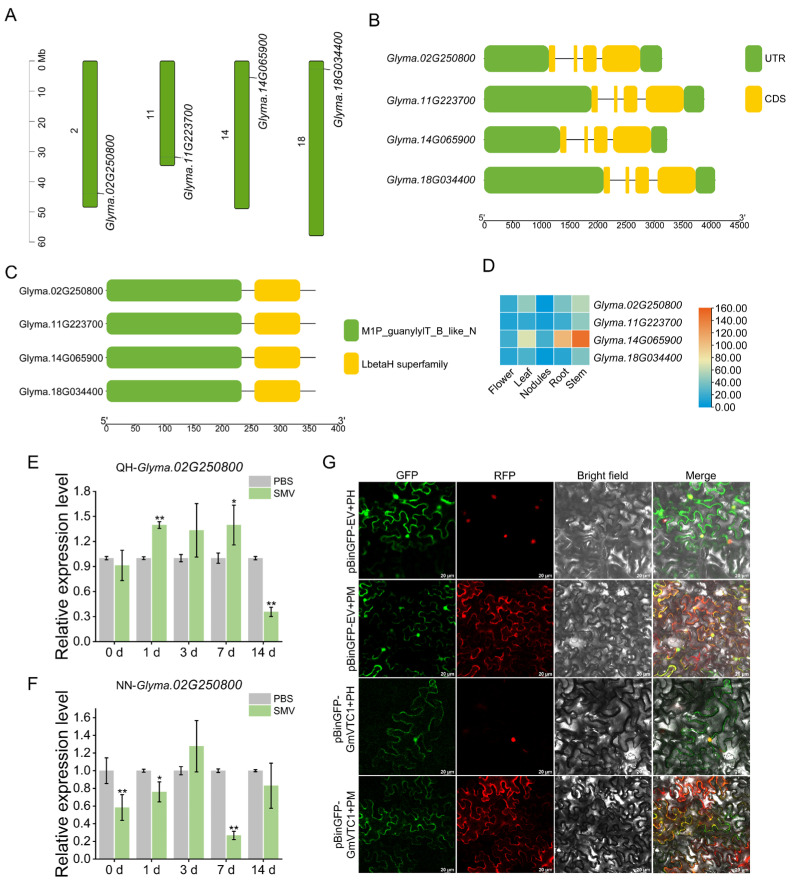
Selection, identification and expression analysis of *GmVTC1*. (**A**,**B**) Chromosomal distribution (**A**) and structural characteristics (**B**) of the soybean VTC1 family genes (**C**) Conserved domains prediction of the four potential GmVTC1 proteins. (**D**) Tissue expression heat map of *GmVTC1* based on FPKM values from the Phytozome database. (**E**,**F**) qRT-PCR analysis of *Glyma.02g250800* expression levels in QH (**E**) and NN (**F**) plants upon SMV induction. The plants inoculated with PBS served as the control group. The *tubulin* was used as the internal reference gene. Values represented the means ± SD of three biological replicates. Differences were analyzed using One-Way ANOVA, *, *p* < 0.05; **, *p* < 0.01. (**G**) Subcellular localization of GmVTC1 observed under the laser confocal microscope. Bar = 20 μm.

Potential *GmVTC1* downstream of *GmCSN5B* were screened through the Phytozome database. Ultimately, four candidate genes (*Glyma.02G250800*, *Glyma.11G223700*, *Glyma.14G065900* and *Glyma.18G034400*) were identified, exhibiting 76.98–78.45% nucleotide sequence identity and 89.75–90.30% amino acid identity with *AtVTC1* (*AT2G39770*) (Figure 4A,B and Figure S2A; Tables S2 and S3). Conserved domain analysis confirmed all four members contain NTP-transferase and Hexapep-domains, indicating high functional conservation (Figure 4C and Figure S2B).

#### 2.4.2. Determine Candidate *GmVTC1* Based on the Expression Under SMV Induction

Based on tissue expression analysis, the four genes were predominantly expressed in nodules, with *Glyma.14G065900* exhibiting higher expression levels in roots and stems (Figure 4D). Following SMV inoculation in QH and NN plants, qRT-PCR revealed that all four genes displayed a regular fluctuating expression pattern in response to SMV infection compared to the controls (Figure 4E,F and Figure S3A–F). Notably, *Glyma.02G250800* showed the most significant differential expression between the two varieties. In QH, its transcripts were upregulated at early infection stages (1–7 dpi), peaked significantly at 1 dpi, and subsequently declined sharply at 14 dpi (Figure 4E); In contrast, relatively basal expression was maintained in NN across the same time course (Figure 4F). Owing to its distinct responsiveness to SMV, *Glyma.02G250800* was preliminarily designated as *GmVTC1* for subsequent functional verification.

#### 2.4.3. Subcellular Localization of GmVTC1

For subcellular localization analysis, the pBinGFP-GmVTC1 recombinant vector was co-infiltrated with either the PM or PH marker into *N. benthamiana* leaves via *Agrobacterium*-mediated transformation. Confocal microscopy assay revealed that GmVTC1 was primarily localized in the cytoplasm and nucleus (Figure 4G).

### 2.5. Interaction Verification Between GmVTC1 and GmCSN5B

**Figure 5:**
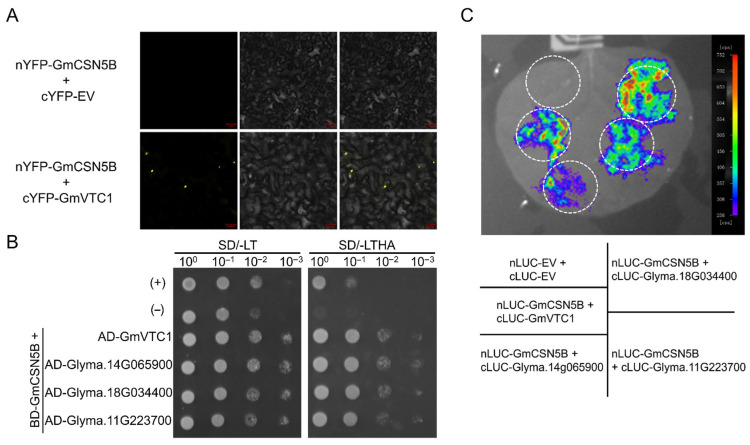
Interaction verification between GmCSN5B and GmVTC1. (**A**–**C**) The interaction between GmCSN5B and GmVTC1 was verified using BiFC (**A**), Y2H (**B**), and LUC (**C**) assays, respectively.

Previous studies have demonstrated that ATCSN5B can interact with AtVTC1 to regulate AsA biosynthesis under oxidative stress [27]. To determine whether a similar interaction exists in soybean, we performed Y2H, BiFC, and LUC assays between GmVTC1 and GmCSN5B. All three assays verified the interaction between the two proteins, and the nuclear localization region was identified as the putative site mediating this interaction (Figure 5A–C). Notably, the other three candidate proteins also exhibited binding affinity with GmCSN5B, indicating functional conservation among VTC1 family members (Figure 5B,C).

### 2.6. Silencing of GmVTC1 Impairs Soybean Resistance to SMV

**Figure 6:**
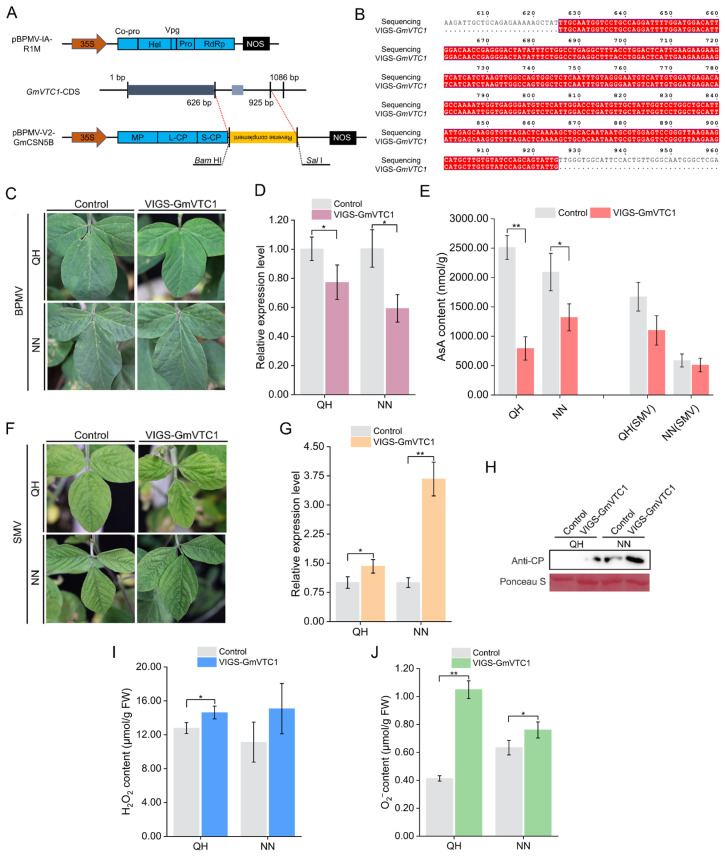
Functional validation of *GmVTC1* using VIGS. (**A**) Schematic illustration of the pBPMV-V2-GmVTC1 vector construction strategy. (**B**) Sequencing analysis of the specific silencing fragment in the recombinant vector. (**C**) Leaf disease phenotypes of VIGS-GmVTC1 and control plants (inoculated with the pBPMV-V2 empty vector) at 7 days after the initial infection of BPMV. (**D**) qRT-PCR analysis of *GmVTC1* silencing efficiency in QH and NN plants at 7 dpi. (**E**) AsA content measurements in QH and NN plants before and after SMV re-inoculation at 10 dpi. (**F**) Upper leaf phenotypes of the above plants after SMV re-inoculation at 10 dpi. (**G**) qRT-PCR analysis of *CP* expression levels in QH and NN plants, with *tubulin* as the internal reference gene. Values represented the means ± SD of three biological replicates. Differences were analyzed using Student’s *t*-test, *, *p* < 0.05; **, *p* < 0.01. (**H**) Western blot analysis of CP accumulation in the upper leaves after SMV re-inoculation, using Ponceau S staining Rubisco subunit as the internal reference protein. (**I**,**J**) Detection of H_2_O_2_ (**I**) and O_2_^•−^ (**J**) content in the upper leaves after SMV re-inoculation. Differences were analyzed using Student’s *t*-test, *, *p* < 0.05; **, *p* < 0.01.

Using the approach described in Section 2.3, a specific fragment targeting *GmVTC1* was designed (Figure 6A,B). The recombinant pBPMV-V2-GmVTC1 construct was mechanically inoculated into QH and NN seedlings to generate *GmVTC1*-silenced plants (Figure 6C). qRT-PCR confirmed efficient knockdown of endogenous *GmVTC1* in both genotypes relative to empty-vector controls (Figure 6D). Concurrent measurements revealed that AsA content was significantly reduced in VIGS-GmVTC1 plants relative to controls (Figure 6E).

Following secondary inoculation with the SC3 strain, VIGS-GmVTC1 plants of QH and NN exhibited obvious leaf curling and mosaic symptoms in their upper leaves compared to controls at 7 dpi (Figure 6F). Viral accumulation analysis revealed significantly higher *CP* gene expression levels in silenced plants through qRT-PCR, which was further confirmed by Western blot showing increased CP accumulation (Figure 6G,H). Consistently, AsA content remained obviously lower in VIGS-GmVTC1 plants post-inoculation (Figure 6E). In addition, elevated accumulation of H_2_O_2_ and O_2_^•−^ was observed in the VIGS-GmVTC1 plants compared with the control plants (Figure 6I,J). These findings collectively suggest that *GmVTC1* likely enhances soybean resistance to SMV, potentially through its regulation of AsA biosynthesis.

## 3. Discussion

Viral infection triggers rapid accumulation of ROS across plant tissues, which participates in defense signaling networks while potentially causing oxidative damage and compromising cellular function when excessively accumulated [30,31]. Notably, Hyodo et al. pioneered the discovery that plant RNA viruses actively harness host ROS-generating machinery to support their robust genome replication, highlighting the dual role of ROS as both defense signals and viral replication facilitators [32]. The SOD system, particularly the CSD, plays a crucial role in maintaining ROS homeostasis by catalyzing the dismutation of O_2_^•−^ into O_2_ and H_2_O_2_ [33]; CCS has been demonstrated to be essential for CSD through copper ion delivery [34]. Building on previous finding that *GmCCS* responds to SMV infection in soybean [13], GmCCS was employed as the bait for Y2H screening of a cDNA library. Subsequent validation through Y2H, BiFC, and LUC assays confirmed its interaction with GmCSN5B (Figure 1C–E). However, no overlap was observed between the bioinformatically predicted GmCCS-interacting proteins and those identified via yeast library screening, a common observation in protein–protein interaction studies [35,36]. This discrepancy mainly stems from the limitations of bioinformatics databases (e.g., reliance on conserved homologous interactions and incomplete soybean-specific interactome data) and the technical specificity of Y2H screening (e.g., dependence on nuclear localization and lack of plant-specific post-translational modifications) [35]. Additionally, the interaction between GmCCS and its partners may be SMV infection-dependent, which cannot be fully simulated by in silico prediction or heterologous yeast systems.

The CSN5 subunit is encoded by two homologous genes (*CSN5A* and *CSN5B*) in many species including Arabidopsis, rice, and soybean [25,29,37]. Notably, CSN5B specifically interacts with VTC1, which is a key enzyme in AsA biosynthesis [27]. In this study, SMV infection triggered opposite expression trends of *GmCSN5B* in the resistant variety QH and the susceptible variety NN (Figure 2E,F), implying its potential role in host defense responses. However, qRT-PCR alone could not fully elucidate its regulatory function. Recent advances in BPMV-induced gene silencing have significantly facilitated functional studies of soybean gene function [38]. For example, silencing the *GmBIR1* gene, which encodes a *BAK1-interacting receptor-like kinase* homolog, was found to enhance SA and H_2_O_2_ accumulation, thereby improving resistance against *Pseudomonas syringae pv.glycinea* (*Psg*) and SMV [39]. Similarly, *GmCSN5B* silencing significantly suppressed viral *CP* expression in both cultivars, suggesting that it negatively regulates soybean resistance to SMV. The undetectable CP in SMV-inoculated QH plants (Figure 3G) may be attributed to the *R_SC3Q_* resistance locus or other constitutive defense [40].

AsA can scavenge ROS directly, protecting plant cells from oxidative stress caused by various environmental factors [41]. In Arabidopsis, seven ascorbate-deficient *vtc* mutants have been identified, corresponding to four *VTC* loci, with *VTC1* encoding GDP-Mannose pyrophosphorylase [42,43]. AsA levels are closely associated with host–pathogen interactions, though their effects vary depending on the pathosystem. For instance, the resistant *Brassica rapa* cultivars accumulated higher AsA levels upon *Turnip mosaic virus* (TuMV) infection [44]. However, AsA deficiency enhanced resistance in Arabidopsis against *P. syringae* and *P. parasitica* [45], suggesting that AsA-mediated defense responses are host- and pathogen-dependent. Among the candidate *GmVTC1* genes analyzed, *Glyma.02G250800* displayed the most pronounced differential expression between QH and NN plants, and was thus preliminarily designated as *GmVTC1* (Figure 4E,F). First, our Y2H, BiFC, and LUC assays confirmed a physical interaction between GmCSN5B and GmVTC1 (Figure 5A–C), which is a prerequisite for the post-translational regulation of GmVTC1 by GmCSN5B. Second, in Arabidopsis, AtCSN5B has been shown to interact with AtVTC1 and regulate its protein stability through ubiquitination-mediated degradation [27], and this interaction is functionally conserved in tomato [28]. Given the high sequence similarity between GmCSN5B and AtCSN5B (Figure 2A), as well as between GmVTC1 and AtVTC1 (89.75–90.30% amino acid identity; Table S2), it is reasonable to infer that GmCSN5B employs a similar mechanism to regulate GmVTC1 protein levels in soybean. Third, our functional data showed that silencing *GmVTC1* increased SMV susceptibility and reduced endogenous AsA levels (Figure 6E–H), indicating an antagonistic functional relationship between GmCSN5B and GmVTC1—consistent with the hypothesis that GmCSN5B negatively regulates GmVTC1 protein accumulation. We acknowledge that direct experimental evidence for GmVTC1 protein level modulation by GmCSN5B is lacking, and this will be the focus of our follow-up studies, including Western blot analysis of GmVTC1 protein abundance in *GmCSN5B*-silenced/overexpressed plants and in vitro ubiquitination assays to verify the regulatory mechanism.

Our study demonstrated that *GmVTC1* silencing significantly increased H_2_O_2_ and O_2_^•−^ accumulation (Figure 6I,J). Given the AsA’s well-established function in ROS scavenging [14,15], it is reasonable to infer that *GmCSN5B* silencing would enhance AsA accumulation (via releasing *GmVTC1* from negative regulation) and thereby reduce ROS levels. Moreover, ROS overaccumulation is typically associated with increased viral susceptibility [30,31], which aligns with our finding that *GmCSN5B* silencing alleviated viral symptoms and reduced CP accumulation.

## 4. Materials and Methods

### 4.1. Plant Materials and Growth Conditions

Soybean cultivars “Qihuang No. 1 (QH)” and “Nannong 1138-2 (NN)” were used in this study. QH is an SMV-resistant cultivar carrying the “*R_SC3Q_*” locus (mapped on chromosome 13) that confers specific resistance to the SC3 strain used in this study [40]. NN is a universally SMV-susceptible cultivar with no known major SMV resistance genes, which exhibits severe mosaic symptoms and high viral accumulation upon SMV infection. The nutrient soil and vermiculite were thoroughly mixed at a 1:1 ratio, hydrated with distilled water and placed in the greenhouse maintained at 25 °C (16 h light)/23 °C (8 h dark). At the VC growth stage, the SC3 strain was inoculated on the true leaves based on the method described by Li et al. [46].

For *Agrobacterium*-mediated transient expression assays, *N. benthamiana* plants were grown in a growth chamber (Model: PGC-150, Yiheng Scientific Instrument Co., Ltd., Shanghai, China) under controlled conditions (16 h light/8 h dark photoperiod, 19–24 °C). The plants were ready for use approximately 6–8 weeks after sowing.

### 4.2. RNA Isolation and qRT-PCR

Total RNA was extracted from soybean leaves using TRIzol reagent (Invitrogen, Carlsbad, CA, USA), followed by first-strand cDNA synthesis using HiScript IV 1st strand cDNA synthesis kit (+ gDNA wiper) (Vazyme, Nanjing, Jiangsu, China). For qRT-PCR assays (Model: CFX96 Touch, Bio-Rad Laboratories, Inc., Hercules, CA, USA), gene-specific primers (Table S4) were designed using Primer Premier 5.0 software, and the primer specificity was initially validated by melting curve analysis (from 60 °C to 95 °C, with a heating rate of 0.5 °C per cycle and fluorescence collection at each step) after PCR amplification. Amplification efficiency of each primer pair was calculated using a standard curve generated by five-fold serial dilutions of mixed cDNA templates, with the efficiency range set at 90–110% and R^2^ ≥ 0.99. qRT-PCR was performed with ChamQ universal SYBR qPCR Master Mix (Vazyme), with the *tubulin* serving as the internal reference gene. The reaction system (20 μL) contained 10 μL of 2 × ChamQ SYBR qPCR Master Mix, 0.4 μL of each forward and reverse primer (10 μM), 2 μL of diluted cDNA template (1:10 *v*/*v*), and 7.2 μL of nuclease-free water. The PCR program was set as follows: 95 °C for 30 s, followed by 40 cycles of 95 °C for 10 s and 60 °C for 30 s. The relative expression levels of the target genes were calculated using the 2^−ΔΔCT^ method.

### 4.3. Knockdown of Candidate Genes via VIGS Based on BPMV

The procedures for constructing gene-silenced vectors and inoculating plasmids have been previously described by Zhang et al. [38]. The pBPMV-IA-V2 vector was digested with *BamH*I (New England Biolabs, Ipswich, MA, USA; the same applies below) and *Sal*I. A 300 bp gene-specific silencing fragment (from its coding sequence) and its reverse complementary sequence was designed using SGN-VIGS database [47], and cloned into the linearized vector with ClonExpress II One Step Cloning Kit (Vazyme) via homologous recombination. The newly constructed plasmids were mixed with the pBPMV-IA-R1M at a 1:1 ratio for inoculation. For the control group, plants were inoculated with the pBPMV-V2 empty vector and pBPMV-R1M vector. The NN plants were initially mechanically inoculated, and after confirmed BPMV infection, the diseased leaves were ground and inoculated onto target plants’ true leaves.

### 4.4. Subcellular Localization Analysis

The subcellular localization of related proteins was predicted using WoLF PSORT [48]. Candidate gene coding sequences were amplified and ligated into the pBinGFP4 vector (digested with *BamH*I and *Kpn*I) for green fluorescent protein (GFP) fusion. The recombinant vectors were transformed into *Agrobacterium* EHA105, and the infection solution (D-(+)-Dextrose 0.5 g, MES 0.6 g, Na_3_PO_4_·12H_2_O 0.12 g, 10 μL 1 mol L^−1^ acetosyringone, in 100 mL ddH_2_O) was prepared. *N. benthamiana* leaves were co-infiltrated with the solutions containing GFP fusion vectors and organelle markers (PM, cell membrane marker; PH, nuclear marker. OD600 = 1.0). After 48 h, confocal imaging (Model: Leica SP8, Leica Microsystems GmbH, Wetzlar, Germany) was performed using excitation/emission wavelengths of 561 nm/575–625 nm (PH/PM) and 488 nm/500–540 nm (GFP).

### 4.5. Yeast Two-Hybrid (Y2H) Assay

The STRING database was utilized to predict potential interacting proteins and networks of GmCCS. Subsequently, we screened a soybean cDNA library using Y2H method in strain AH109, following previous protocols [11]. The constructed pGBK-GmCCS vector (pGBKT7 was digested with *Nde*I and *Not*I) was used as the bait. We then used Yeast plasmid extraction kit (Solarbio, Beijing, China) to extract positive yeast colonies grew on SD/-Leu/-Trp/-His/-Ade selective plates. These plasmids were transformed into *E. coli* DH5α competent cells and cultured on LB medium supplemented with 50 μg mL^−1^ ampicillin. PCR detection was performed using AD-cexu-F/R primers, and sequencing results were aligned against the Phytozome database.

For pairwise interaction validation, the recombinant pGBKT7 and pGADT7 (prey) vectors (digested with *Nde*I and *BamH*I) were co-transformed into AH109 yeast cells. Serial dilutions (10×, 100×, 1000×) of yeast cultures were spotted onto SD/-Leu/-Trp/-His/-Ade and SD/-Leu/-Trp selective plates, which were incubated at 30 °C for 5 days before observation.

### 4.6. Bimolecular Fluorescence Complementation (BiFC) and Dual-Luciferase (LUC) Assays

The recombinant vectors (p1300-nLUC and p1300-cLUC were digested with *Kpn*I and *Sal*I; pGD-c-nYFP and pGD-c-cYFP were digested with *Sma*I and *EcoR*I) were transformed into *Agrobacterium* EHA105. Following the preparation of infection solution (as in Section 4.4), transient expression was performed in *N. benthamiana* via leaf infiltration. For LUC assays, 0.3 mg mL^−1^ D-fluorescein potassium solution (Solarbio) was sprayed onto the infiltration areas. After 5 min dark adaptation, luminescence was captured using an in vivo imaging system (Berthold LB 985); for BiFC assays, confocal microscope was employed with yellow fluorescent protein (YFP) excitation at 514 nm and emission detection at 520–550 nm.

### 4.7. Determination of AsA Content

Ascorbic acid content assay kit (Solarbio) was used to detect AsA content in soybean leaves. For extraction, samples (0.1 g) were homogenized with 1 mL extraction buffer. The supernatant (100 μL) or AsA standard (400 μmol L^−1^, 100 μL) was mixed with 800 μL Reagent II and 100 μL Reagent III. Absorbance at 265 nm was recorded by SpectraMax i3x microplate reader (Molecular Devices LLC, San Jose, CA, USA) at 10 s (A1/A3 for standard/sample) and 130 s (A2/A4). Calculated ΔA standard tube = A1 − A2, ΔA determination tube = A3 − A4; AsA content (nmol g^−1^ FW) = 400 × ΔA determination tube/ΔA standard tube/Weight.

### 4.8. Statistical Analysis

All experimental data were presented as the mean ± standard deviation (SD) of at least three independent biological replicates with three technical replicates each. Normality and homogeneity of variance were verified before statistical analysis. Student’s *t*-test was used for comparisons between two groups (e.g., control vs. VIGS plants), and One-Way analysis of variance (One-Way ANOVA) followed by Duncan’s multiple range test was used for comparisons among more than two groups (e.g., gene expression at different time points post-SMV inoculation). All statistical analyses were performed using Origin 2024 software, and differences were considered statistically significant at “*p* < 0.05” and extremely significant at “*p* < 0.01”.

## 5. Conclusions

In conclusion, the *GmCCS-GmCSN5B-GmVTC1* pathway was identified as a crucial regulator of soybean–SMV interactions, with *GmCSN5B* acting as a negative regulator and *GmVTC1* as a positive regulator via the modulation of AsA levels. These findings advance our understanding of soybean antiviral defense mechanisms and offer potential molecular targets for resistance breeding programs.

## Figures and Tables

**Table 1:** Summary table of GmCCS-interacting proteins selected from yeast library.

Bait Protein	Potential Interacting Proteins	Functional Annotation
GmCCS	Glyma.03G028800	METHIONINE AMINOPEPTIDASE 1
	Glyma.06G076000	COP9 SIGNALOSOME COMPLEX SUBUNIT 5
	Glyma.06G019800	MULTI-COPPER OXIDASE TYPE I FAMILY PROTEIN-RELATED
	Glyma.06G020400	PLASTOCYANIN MAJOR ISOFORM, CHLOROPLASTIC-RELATED
	Glyma.08G196400	PWWP domain (PWWP)
	Glyma.09G117100	CALCIUM-DEPENDENT LIPID-BINDING DOMAIN-CONTAINING
	Glyma.09G240600	VOLTAGE-DEPENDENT ANION-SELECTIVE CHANNEL
	Glyma.11G098700	PEPTIDYL-PROLYL CIS-TRANS ISOMERASE CYP19-1
	Glyma.11G162500	TRANSCRIPTION FACTOR BHLH35-LIKE
	Glyma.15G183100	RED PROTEIN IK FACTOR CYTOKINE IK
	Glyma.17G095200	DYNEIN LIGHT CHAIN

## Data Availability

The original contributions presented in this study are included in the article. Further inquiries can be directed to the corresponding author.

## Supplementary Materials

The following supporting information can be downloaded at: https://www.mdpi.com/article/10.3390/plants15071020/s1, Figure S1. Phylogenetic, tissue expression and gene structure analyses of *GmCSN5B*. (A) Phylogenetic tree of CSN5B from different species, constructed by the Neighbor-Joining method in MEGA 7.0. The bootstrap replication was set to 1000 replicates, and other parameters were set to default values. (B) Tissue expression profile of *GmCSN5B* based on FPKM values downloaded from the Phytozome database. (C) The gene structure analysis of *GmCSN5B* using TBtools-II v2.390. Figure S2. Characterization of the potential *VTC1* genes in soybean. (A) The amino acid sequence alignment of the four *VTC1* genes identified in soybean and the homologous gene (*At2G39770*) in *Arabidopsis thaliana*. (B) Prediction of conserved motifs in 4 *VTC1* genes. (C) Prediction of *cis*-acting elements related to stress in the upstream promoters of four the *VTC1* genes. Figure S3. Detection of *VTC1* gene expression levels in soybean induced by SMV. (A–F) qRT-PCR analysis was performed to detect the expression levels of *Glyma.11G223700*, *Glyma.14G065900* and *Glyma.18G034400* in QH and NN plants upon SMV induction, and plants inoculated with PBS served as the control group. The *tubulin* was used as the internal reference gene. Values represented the means ± SD of three biological replicates. Differences were analyzed using Student’s *t*-test, *, *p* < 0.05; **, *p* < 0.01. Table S1. The interactive proteins predicted by STRING website. Table S2. Analysis of the consistency and similarity of the amino acid sequences between candidate GmVTC1 and AtVTC1. Table S3. Analysis of the consistency rate of four candidate *GmVTC1* and *AtVTC1* gene coding sequences. Table S4. Primers used in this study.

## References

[1] Hajimorad M.R., Domier L.L., Tolin S.A., Whitham S.A., Saghai Maroof M.A. (2018). *Soybean mosaic virus*: A successful potyvirus with a wide distribution but restricted natural host range. Mol. Plant Pathol..

[2] Usovsky M., Chen P., Li D., Wang A., Shi A., Zheng C., Shakiba E., Lee D., Canella Vieira C., Lee Y.C. (2022). Decades of genetic research on *Soybean mosaic virus* resistance in soybean. Viruses.

[3] Widyasari K., Alazem M., Kim K.H. (2020). Soybean resistance to *Soybean mosaic virus*. Plants.

[4] Hayes A., Jeong S., Gore M., Yu Y., Buss G., Tolin S., Maroof M. (2004). Recombination within a nucleotide-binding-site/leucine-rich-repeat gene cluster produces new variants conditioning resistance to *Soybean mosaic virus* in soybeans. Genetics.

[5] Suh S., Bowman B., Jeong N., Yang K., Kastl C., Tolin S., Maroof M., Jeong S. (2011). The *Rsv3* locus conferring resistance to *Soybean Mosaic Virus* is associated with a cluster of coiled-coil nucleotide-binding leucine-rich repeat genes. Plant Genome.

[6] Maroof M., Tucker D., Skoneczka J., Bowman B., Tripathy S., Tolin S. (2010). Fine mapping and candidate gene discovery of the *Soybean mosaic virus* resistance gene, *Rsv4*. Plant Genome.

[7] Li C., Adhimoolam K., Yuan Y., Yin J., Ren R., Yang Y., Zhi H. (2017). Identification of candidate genes for resistance to *Soybean mosaic virus* strain SC3 by using fine mapping and transcriptome analyses. Crop Pasture Sci..

[8] Wang D., Ma Y., Liu N., Yang Z., Zheng G., Zhi H. (2011). Fine mapping and identification of the soybean *R_SC4_* resistance candidate gene to *Soybean mosaic virus*. Plant Breed..

[9] Zhang Y., Gao P., Yuan J. (2010). Plant protein-protein interaction network and interactome. Curr. Genom..

[10] Struk S., Jacobs A., Martín-Fontecha E.S., Gevaert K., Cubas P., Goormachtig S. (2018). Exploring the protein-protein interaction landscape in plants. Plant Cell Environ..

[11] Hu T., Luan H., Wang L., Ren R., Sun L., Yin J., Liu H., Jin T., Li B., Li K. (2023). *Soybean mosaic virus* 6K1 interactors screening and GmPR4 and GmBI1 function characterization. Int. J. Mol. Sci..

[12] Fang L., Geng C., Wei X., Dong C., Pang J., Yan Z., Jiang J., Tian Y., Li X. (2024). Potato virus Y viral protein 6K1 inhibits the interaction between defense proteins during virus infection. Plant Physiol..

[13] Li B., Wang L., Qian X., Liu H., Jin T., Yin J., Hu T., Liu M., Guo D., Li K. (2025). Gma-miR398c/d negatively regulates soybean resistance to *Soybean mosaic virus* by targeting SOD family genes. Crop J..

[14] Smirnoff N., Wheeler G.L. (2010). Ascorbic acid in plants: Biosynthesis and function. Crit. Rev. Plant Sci..

[15] Viviani A., Fambrini M., Giordani T., Pugliesi C. (2021). L-Ascorbic acid in plants: From biosynthesis to its role in plant development and stress response. Agrochimica.

[16] Hemavathi, Upadhyaya C.P., Akula N., Young K.E., Chun S.C., Kim D.H., Park S.W. (2009). Enhanced ascorbic acid accumulation in transgenic potato confers tolerance to various abiotic stresses. Biotechnol. Lett..

[17] Khazaei Z., Esmaielpour B., Estaji A. (2020). Ameliorative effects of ascorbic acid on tolerance to drought stress on pepper (*Capsicum annuum* L) plants. Physiol. Mol. Biol. Plants.

[18] Fujiwara A., Togawa S., Hikawa T., Matsuura H., Masuta C., Inukai T. (2016). Ascorbic acid accumulates as a defense response to *Turnip mosaic virus* in resistant *Brassica rapa cultivars*. J. Exp. Bot..

[19] Kumari R., Kumar S., Singh L., Hallan V. (2016). Movement protein of *Cucumber mosaic virus* associates with Apoplastic Ascorbate Oxidase. PLoS ONE.

[20] Agius F., González-Lamothe R., Caballero G.L., Muñoz-Blanco J., Botella M.A., Valpuesta V. (2003). Engineering increased vitamin C levels in plants by overexpression of a D-galacturonic acid reductase. Nat. Biotechnol..

[21] Lorence A., Chevone B.I., Mendes P., Nessler C.L. (2004). myo-inositol oxygenase offers a possible entry point into plant ascorbate biosynthesis. Plant Physiol..

[22] Wheeler G.L., Jones M.A., Smirnoff N. (1998). The biosynthetic pathway of vitamin C in higher plants. Nature.

[23] Ishikawa T., Shigeoka S. (2014). Recent advances in ascorbate biosynthesis and the physiological significance of ascorbate peroxidase in photosynthesizing organisms. Biosci. Biotechnol. Biochem..

[24] Wei N., Deng X.W. (2003). The COP9 signalosome. Annu. Rev. Cell Dev. Biol..

[25] Kwok S.F., Solano R., Tsuge T., Chamovitz D.A., Ecker J.R., Matsui M., Deng X.W. (1998). Arabidopsis homologs of a c-Jun coactivator are present both in monomeric form and in the COP9 complex, and their abundance is differentially affected by the pleiotropic *cop/det/fus* mutations. Plant Cell.

[26] Gusmaroli G., Feng S., Deng X.W. (2004). The *Arabidopsis* CSN5A and CSN5B subunits are present in distinct COP9 signalosome complexes, and mutations in their JAMM Domains exhibit differential dominant negative effects on development. Plant Cell.

[27] Wang J., Yu Y., Zhang Z., Quan R., Zhang H., Ma L., Deng X., Huang R. (2013). *Arabidopsis* CSN5B interacts with VTC1 and modulates ascorbic acid synthesis. Plant Cell.

[28] Li Y., Chu Z., Luo J., Zhou Y., Cai Y., Lu Y., Xia J., Kuang H., Ye Z., Ouyang B. (2017). The C2H2 zinc-finger protein SlZF3 regulates AsA synthesis and salt tolerance by interacting with CSN5B. Plant Biotechnol. J..

[29] Mo X., Zhang M., Zhang Z., Lu X., Liang C., Tian J. (2021). Phosphate (Pi) starvation up-regulated GmCSN5A/B participates in anthocyanin synthesis in soybean (*Glycine max*) dependent on Pi availability. Int. J. Mol. Sci..

[30] Xu Y., Zhang S., Zhang M., Jiao S., Guo Y., Jiang T. (2024). The role of reactive oxygen species in plant-virus interactions. Plant Cell Rep..

[31] Mittler R., Zandalinas S.I., Fichman Y., Van Breusegem F. (2022). Reactive oxygen species signalling in plant stress responses. Nat. Rev. Mol. Cell Biol..

[32] Hyodo K., Hashimoto K., Kuchitsu K., Suzuki N., Okuno T. (2017). Harnessing host ROS-generating machinery for the robust genome replication of a plant RNA virus. Proc. Natl. Acad. Sci. USA.

[33] McCord J.M., Fridovich I. (1969). Superoxide dismutase. J. Biol. Chem..

[34] Cohu C.M., Abdel-Ghany S.E., Gogolin Reynolds K.A., Onofrio A.M., Bodecker J.R., Kimbrel J.A., Niyogi K.K., Pilon M. (2009). Copper delivery by the copper chaperone for chloroplast and cytosolic copper/zinc-superoxide dismutases: Regulation and unexpected phenotypes in an *Arabidopsis* Mutant. Mol. Plant.

[35] Vidal M., Legrain P. (2014). Yeast two-hybrid: A powerful tool for systems biology. FEBS Lett..

[36] De Las Rivas J., Fontanillo C. (2010). Protein-protein interaction databases: A survey. Nucleic Acids Res..

[37] Wang J., Zhang C., Li H., Xu Y., Zhang B., Zheng F., Zhao B., Zhang H., Zhao H., Liu B. (2023). OsJAB1 positively regulates ascorbate biosynthesis and negatively regulates salt tolerance due to inhibiting early-stage salt-induced ROS accumulation in rice. Plants.

[38] Zhang C., Bradshaw J.D., Whitham S.A., Hill J.H. (2010). The development of an efficient multipurpose *Bean pod mottle virus* viral vector set for foreign gene expression and RNA silencing. Plant Physiol..

[39] Liu D., Lan H., Masoud H.S., Ye M., Dai X., Zhong C., Tian S., Liu J. (2022). Silencing *GmBIR1* in soybean results in activated defense responses. Int. J. Mol. Sci..

[40] Zheng G., Yang Y., Ma Y., Yang X., Chen S., Ren R., Wang D., Yang Z., Zhi H. (2014). Fine mapping and candidate gene analysis of resistance gene *Rsc_3Q_* to *Soybean mosaic virus* in Qihuang 1. J. Integr. Agr..

[41] Hossain M.A., Munné-Bosch S., Burritt D.J., Diaz-Vivancos P., Fujita M., Lorence A. (2017). Ascorbic Acid in Plant Growth, Development and Stress Tolerance.

[42] Conklin P.L., Saracco S.A., Norris S.R., Last R.L. (2000). Identification of ascorbic acid-deficient *Arabidopsis thaliana* mutants. Genetics.

[43] Conklin P.L., Norris S.R., Wheeler G.L., Williams E.H., Smirnoff N., Last R.L. (1999). Genetic evidence for the role of GDP-mannose in plant ascorbic acid (vitamin C) biosynthesis. Proc. Natl. Acad. Sci. USA.

[44] Fujiwara A., Shimura H., Masuta C., Sano S., Inukai T. (2013). Exogenous ascorbic acid derivatives and dehydroascorbic acid are effective antiviral agents against *Turnip mosaic virus* in *Brassica rapa*. J. Gen. Plant Pathol..

[45] Barth C., Moeder W., Klessig D.F., Conklin P.L. (2004). The timing of senescence and response to pathogens is altered in the ascorbate-deficient *Arabidopsis* mutant *vitamin c-1*. Plant Physiol..

[46] Li K., Yang Q., Zhi H., Gai J. (2010). Identification and distribution of *Soybean mosaic virus* strains in southern China. Plant Dis..

[47] Fernandez-Pozo N., Rosli H.G., Martin G.B., Mueller L.A. (2015). The SGN VIGS tool: User-friendly software to design virus-induced gene silencing (VIGS) constructs for functional genomics. Mol. Plant.

[48] Horton P., Park K.J., Obayashi T., Fujita N., Harada H., Adams-Collier C.J., Nakai K. (2007). WoLF PSORT: Protein localization predictor. Nucleic Acids Res..

