# Supplementary material for: Characterization of the Soybean GmCCS-GmCSN5B-GmVTC1 Pathway and Its Functional Roles Under Soybean mosaic virus Infection
Source: Plants (Basel). 2026 Mar 26;15(7):1020. doi: 10.3390/plants15071020 (PMC13075224; doi:10.3390/plants15071020)
Supplement: Supplementary file 1 [file plants-15-01020-s001.zip › Supplementary files.pdf]

SUPPLEMENTARY FILE

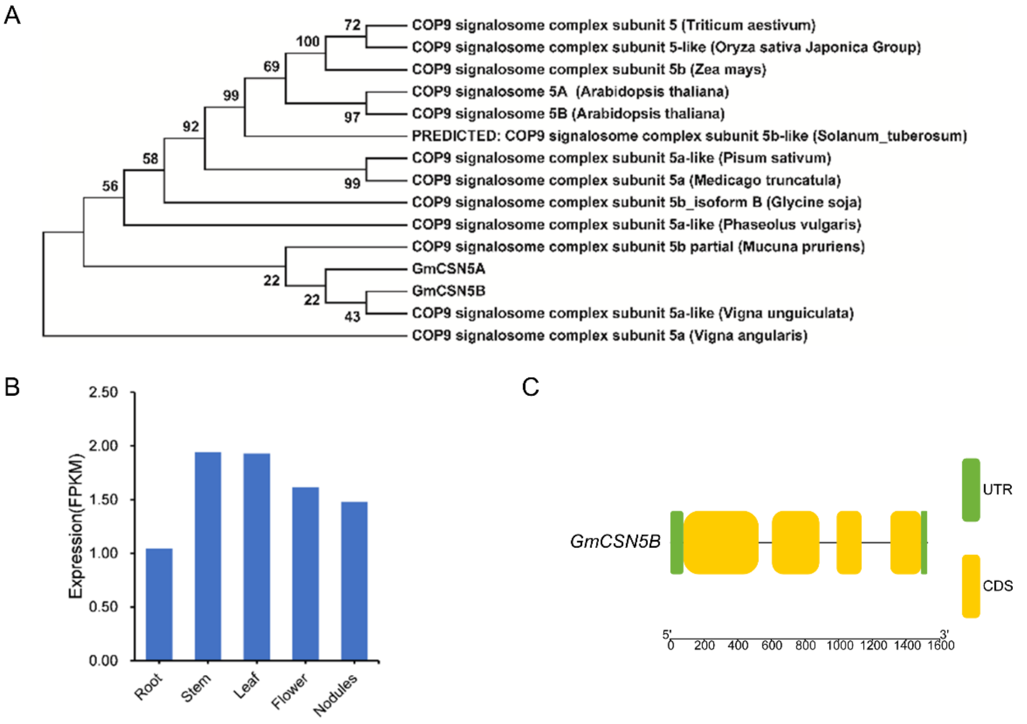

Figure S1. Phylogenetic, tissue expression and gene structure analyses of *GmCSN5B*. (**A**) Phylogenetic tree of CSN5B from different species, constructed by the Neighbor-Joining method in MEGA 7.0. The bootstrap replication was set to 1000 replicates, and other parameters were set to default values. (**B**) Tissue expression profile of *GmCSN5B* based on FPKM values downloaded from the Phytozome database. (**C**) The gene structure analysis of *GmCSN5B* using TBtools.

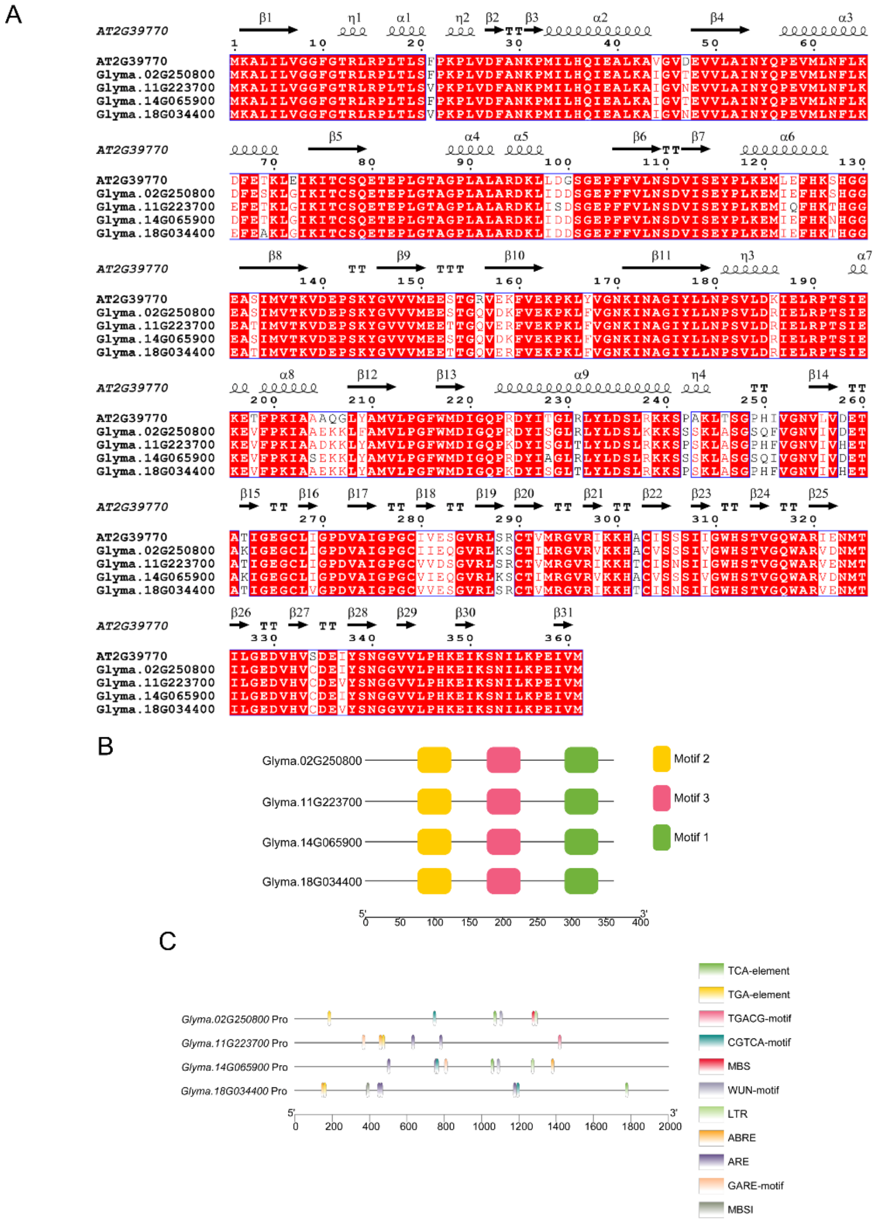

Figure S2. Characterization of the potential *VTC1* genes in soybean. (**A**) The amino acid sequence alignment of the four *VTC1* genes identified in soybean and the homologous gene (*At2G39770*) in *Arabidopsis thaliana*. (**B**) Prediction of conserved motifs in 4 *VTC1* genes. (**C**) Prediction of *cis*-acting elements related to stress in the upstream promoters of four the VTC1 genes.

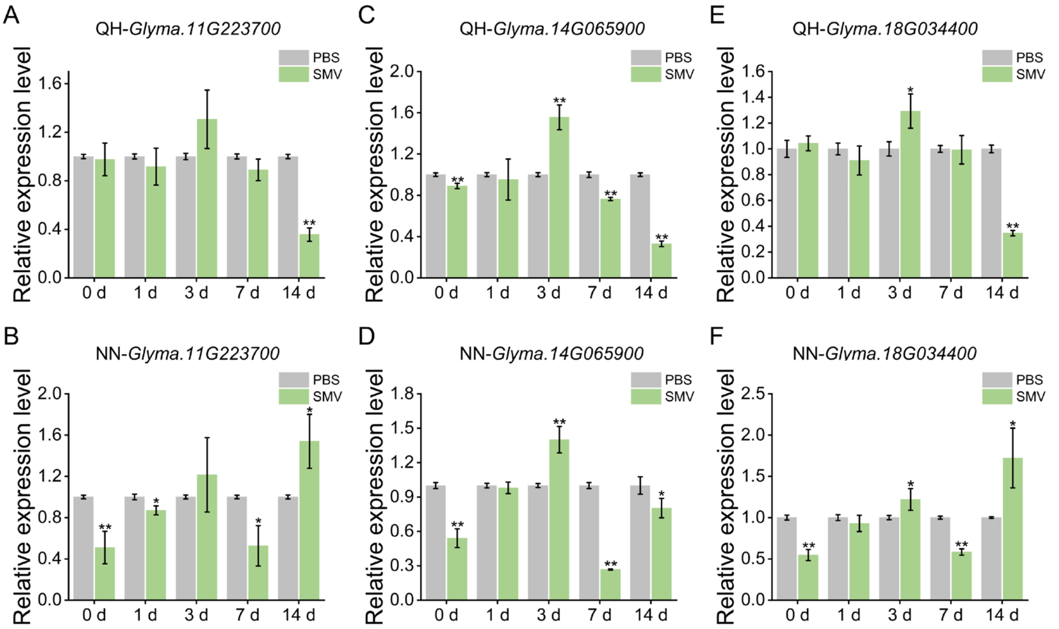

Figure S3. Detection of *VTC1* gene expression levels in soybean induced by SMV. (A–F) qRT-PCR analysis was performed to detect the expression levels of *Glyma.11G223700*, *Glyma.14G065900* and *Glyma.18G034400* in QH and NN plants upon SMV induction, and plants inoculated with PBS served as the control group. The *tubulin* was used as the internal reference gene. Values represented the means ± SD of three biological replicates. Differences were analyzed using Student's *t*-test, *, $P < 0.05$; **, $P < 0.01$.

Table S1. The interactive proteins predicted by STRING database

Bait protein	Predicted interactive protein	Functional Annotation
GmCCS	Glyma.11G192700<br>Glyma.12G081300<br>Glyma.12G178800	SUPEROXIDE DISMUTASE [CU-ZN] 2, CHLOROPLASTIC
	Glyma.06G056300<br>Glyma.16G088300<br>Glyma.09G052000	E1-E2 ATPase (E1-E2_ATPase) // Heavy-metal-associated domain (HMA) // haloacid dehalogenase-like hydrolase (HAD)
	Glyma.16G153900	SUPEROXIDE DISMUTASE [CU-ZN] 3
	Glyma.03G242900<br>Glyma.19G240400	SUPEROXIDE DISMUTASE CU-ZN -RELATED
	Glyma.09G218700	EXPRESSED PROTEIN-RELATED

Table S2. Analysis of the consistency and similarity of the amino acid sequences between candidate GmVTC1 and AtVTC1

	AT2G39770	Glyma.02G250800	Glyma.11G223700	Glyma.14G065900	Glyma.18G034400
AT2G39770	—	—	—	—	—
Glyma.02G250800	89.75%/94.74%	—	—	—	—
Glyma.11G223700	90.03%/95.29%	90.30%/96.12%	—	—	—
Glyma.14G065900	90.03%/94.18%	97.78%/98.89%	90.58%/95.01%	—	—
Glyma.18G034400	90.30%/95.29%	91.14%/96.12%	98.34%/99.45%	91.14%/95.01%	—

Note: The value before "/" represents consistency rate, while the value after "/" represents similarity rate.

Table S3. Analysis of the consistency rate of four candidate *GmVTC1* and *AtVTC1* gene coding sequences

	AT2G39770	*Glyma.02G250800*	*Glyma.11G223700*	*Glyma.14G065900*	*Glyma.18G034400*
AT2G39770	—	—	—	—	—
Glyma.02G250800	76.98%	—	—	—	—
Glyma.11G223700	78.45%	87.48%	—	—	—
Glyma.14G065900	77.16%	96.96%	87.02%	—	—
Glyma.18G034400	78.08%	87.94%	97.79%	87.38%	—

Table S4. Primers used in this study

primer name	Sequence
pBin-Gm06g076000-F	GACGAGCTGTACAAGGGTACCATGCAAGGCAAAGAAATGGAAGGG
pBin-Gm06g076000-R	TCATCTAGAGGATCCGTCGACACTTTCAATCATTGGTTCGGGACCAG
pBin-Gm02g250800-F	GACGAGCTGTACAAGGGTACCATGAAGGCATTGATTCTGGTTGGG
pBin-Gm02g250800-R	TCATCTAGAGGATCCGTCGACCATGACAATCTCCGGCTTCAG
pBin-cexu-F	CCAGGGTTTTCCCAGTCA
pBin-cexu-R	GCACCCCAGGCTTTACAC
VIGS-Gm06g076000-F	GAATCCTCTGCATGAGGATCCTCCTCAATCTTATTGAGAGGAATAGTC
VIGS-Gm06g076000-R	CTCTCGAGGCCTGGAGTCGACTACGAGTACATGGTCGATTATTCCC
VIGS-Gm02g250800-F	GAATCCTCTGCATGAGGATCCCAATACTGCTGGATACACAAGC
VIGS-Gm02g250800-R	CTCTCGAGGCCTGGAGTCGACTTGCAATGGTCCTGCCAGG
BPMV-R2-C2F	TGACATTCTCCTGGGAATTTCCC
BPMV-R2-C2R	CACACTTCACACATCATTACGAC
Gm06g076000-qF	CACTAATAGCACCCACGCCT
Gm06g076000-qR	GTCCTTGATGACCTGCGACA
Gm02g250800-qF	AATAATGCGTGGAGTCCGGG
Gm02g250800-qR	AACAGTGGAATGCCACCCAA
Gm14g065900-qF	AACCAAGGTTGACGAGCCAT
Gm14g065900-qR	CCTGGCAGGACCATTGCATA
Gm18g034400-qF	TGTTGAGTCAGGTGTCAGGC
Gm18g034400-qR	ACAGTGGAATGCCACCCAAT
Gm11g223700-qF	TGTTGACTCAGGTGTCAGGC
Gm11g223700-qF	ACAGTGGAATGCCACCCAAT
SMV-CP-qF	TTCTGAAAGTCCGTATATGCCTAG
SMV-CP-qR	GCCTTTCAGTATTTTCGGAGTT
Tubulin-qF	GGAGTTCACAGAGGCAGAG
Tubulin-qR	CACTTACGCATCACATAGCA
BD-GmCCS-F	CTCAGAGGAGGACCTGCATATGATGGCATTTCTGAGGTCAATAGCAAC
BD-GmCCS-R	GTTATGCTAGTTATGCGGCCGCGACCTTGCTAGTAACAAAATCTGTATCCG
BD-Gm06g076000-F	CTCAGAGGAGGACCTGCATATGATGCAAGGCAAAGAAATGGAAGGG

BD-Gm06g076000-R	GTTATGCTAGTTATGCGGCCGCACTTTCAATCATTGGTTCGGGACCAG
AD-Gm06g076000-F	GTACCAGATTACGCTCATATGATGCAAGGCAAAGAAATGGAAGGG
AD-Gm06g076000-R	CAGCTCGAGCTCGATGGATCCACTTTCAATCATTGGTTCGGGACCAG
AD-Gm02g250800-F	GTACCAGATTACGCTCATATGATGAAGGCATTGATTCTGGTTGGG
AD-Gm02g250800-R	CAGCTCGAGCTCGATGGATCCCATGACAATCTCCGGCTTCAG
AD-Gm14g065900-F	GTACCAGATTACGCTCATATGATGAAGGCATTGATTCTGGTTGGG
AD-Gm14g065900-R	CAGCTCGAGCTCGATGGATCCCATGACAATCTCCGGCTTCAG
AD-Gm18g034400-F	GTACCAGATTACGCTCATATGATGAAGGCACTAATTCTTGTTGGAGG
AD-Gm18g034400-R	CAGCTCGAGCTCGATGGATCCCATGACAATCTCTGGCTTTAGAATG
AD-Gm11g223700-F	GTACCAGATTACGCTCATATGATGAAGGCACTAATTCTTGTTGGAGG
AD-Gm11g223700-R	CAGCTCGAGCTCGATGGATCCCATGACTATCTCTGGCTTCAGAATG
BD-cexu-F	GTCTGAAGAACAACTGGGAGTG
BD-cexu-R	CTGAGAAAGCAACCTGACCTAC
AD-cexu-F	CGTATAACGCGTTTGGAATCAC
AD-cexu-R	CTATAGATCAGAGGTTACATGGCC
n/cYFP-GmCCS-F	TCTAGAACTAGTGGATCCATGGCATTTCTGAGGTCAATAGC
n/cYFP-GmCCS-R	GATAAGCTTGATATCGAATTCGACCTTGCTAGTAACAAAATCTG
c/nYFP-Gm06g076000-F	TCTAGAACTAGTGGATCCATGCAAGGCAAAGAAATGGAAGGG
c/nYFP-Gm06g076000-R	GATAAGCTTGATATCGAATTCTTAACTTTCAATCATTGGTTCGGGACCAG
cYFP-Gm02g250800-F	TCTAGAACTAGTGGATCCATGAAGGCATTGATTCTGGTTGGG
cYFP-Gm02g250800-R	GATAAGCTTGATATCGAATTCTCACATGACAATCTCCGGCTTCAG
cYFP-cexu-F	GAGCTGTACAAGACTAGTGACTAC
cYFP-cexu-R	ACATGCTTAACGTAATTCAACAG
nYFP-cexu-F	ACATCGAGGACACTAGTGAC
nYFP-cexu-R	ACATGCTTAACGTAATTCAACAG
nLUC-GmCCS-F	ACGGGGGACGAGCTCGGTACCATGGCATTTCTGAGGTCAATAGC

nLUC-GmCCS-R	CGCGTACGAGATCTGGTCGACGACCTTGCTAGTAACAAAATCTG
cLUC-GmCCS-F	TACGCGTCCCGGGGCGGTACCATGGCATTTCTGAGGTCAATAGC
cLUC-GmCCS-R	ACGAAAGCTCTGCAGGTCGACGACCTTGCTAGTAACAAAATCTG
cluc-Gm06g076000-F	TACGCGTCCCGGGGCGGTACCATGCAAGGCAAAGAAATGGAAGGG
cluc-Gm06g076000-R	ACGAAAGCTCTGCAGGTCGACTTAACTTTCAATCATTGGTTCGGGACCAG
cluc-Gm02g250800-F	TACGCGTCCCGGGGCGGTACCATGAAGGCATTGATTCTGGTTGGG
cluc-Gm02g250800-R	ACGAAAGCTCTGCAGGTCGACTCACATGACAATCTCCGGCTTCAG
cLUC-cexu-F	TTACCGGAAAACTCGACGCAAG
cLUC-cexu-R	CTGGTGTGTGCGCAATGAAAC
nLUC-cexu-F	GACGCACAATCCCACTATCC
nLUC-cexu-R	TTCCAGCGGATAGAATGGCG
