# Supplementary figures and images for: Characterization of the Soybean GmCCS-GmCSN5B-GmVTC1 Pathway and Its Functional Roles Under Soybean mosaic virus Infection
Source: Plants (Basel). 2026 Mar 26;15(7):1020. doi: 10.3390/plants15071020 (PMC13075224; doi:10.3390/plants15071020)

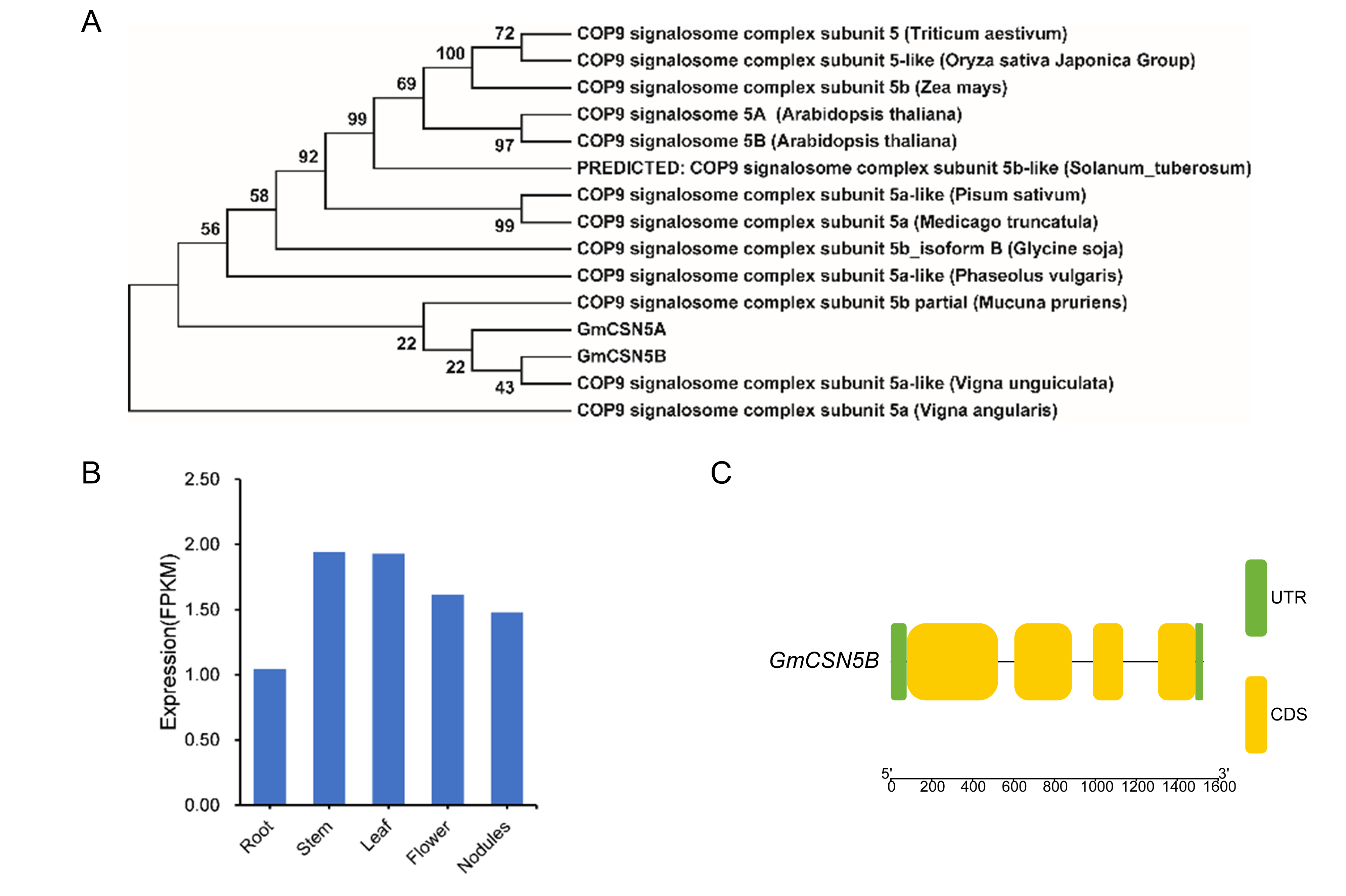

Supplement: Supplementary file 1 [file plants-15-01020-s001.zip › Figures/Figure S1.tif]

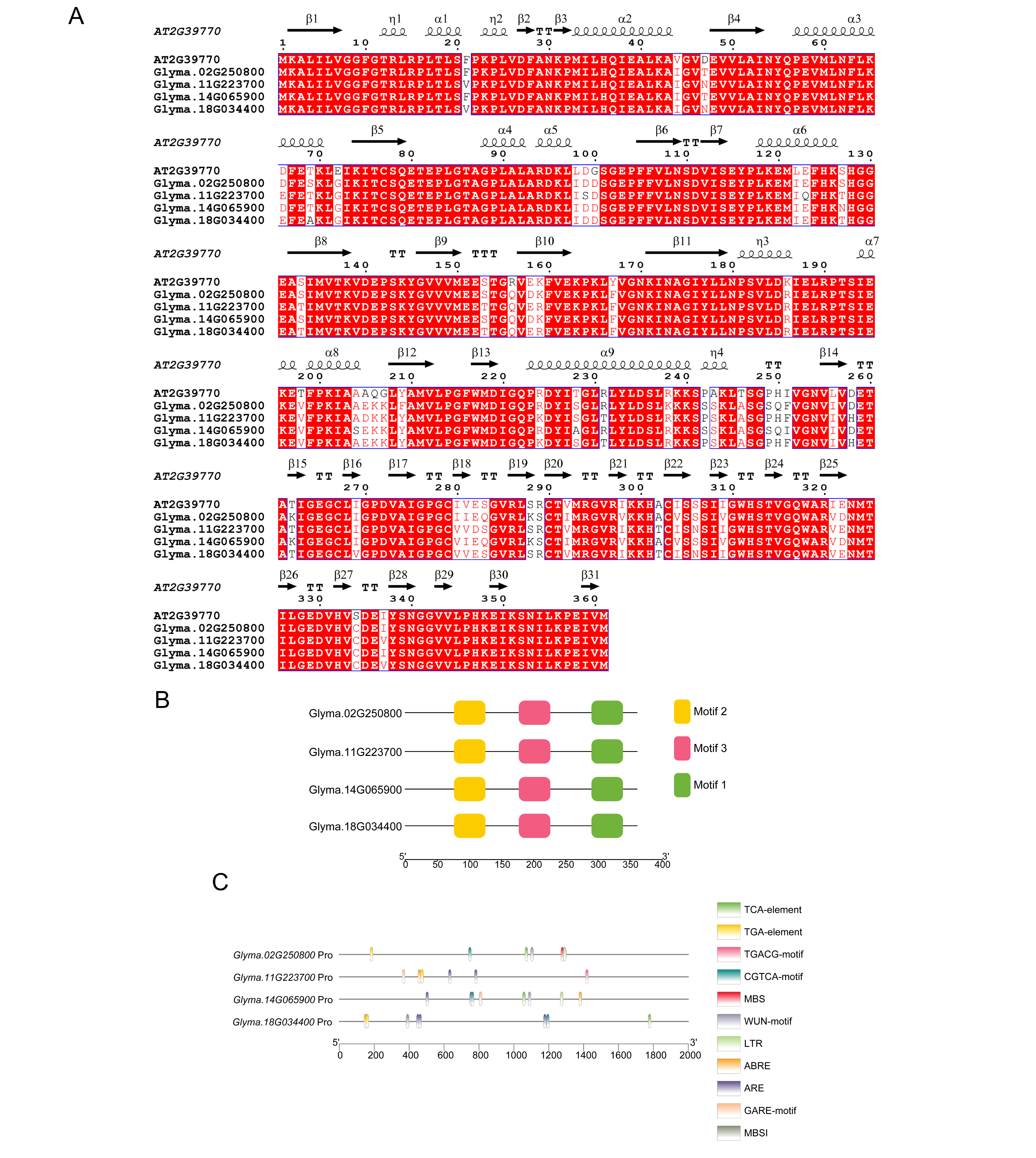

Supplement: Supplementary file 1 [file plants-15-01020-s001.zip › Figures/Figure S2.tif]

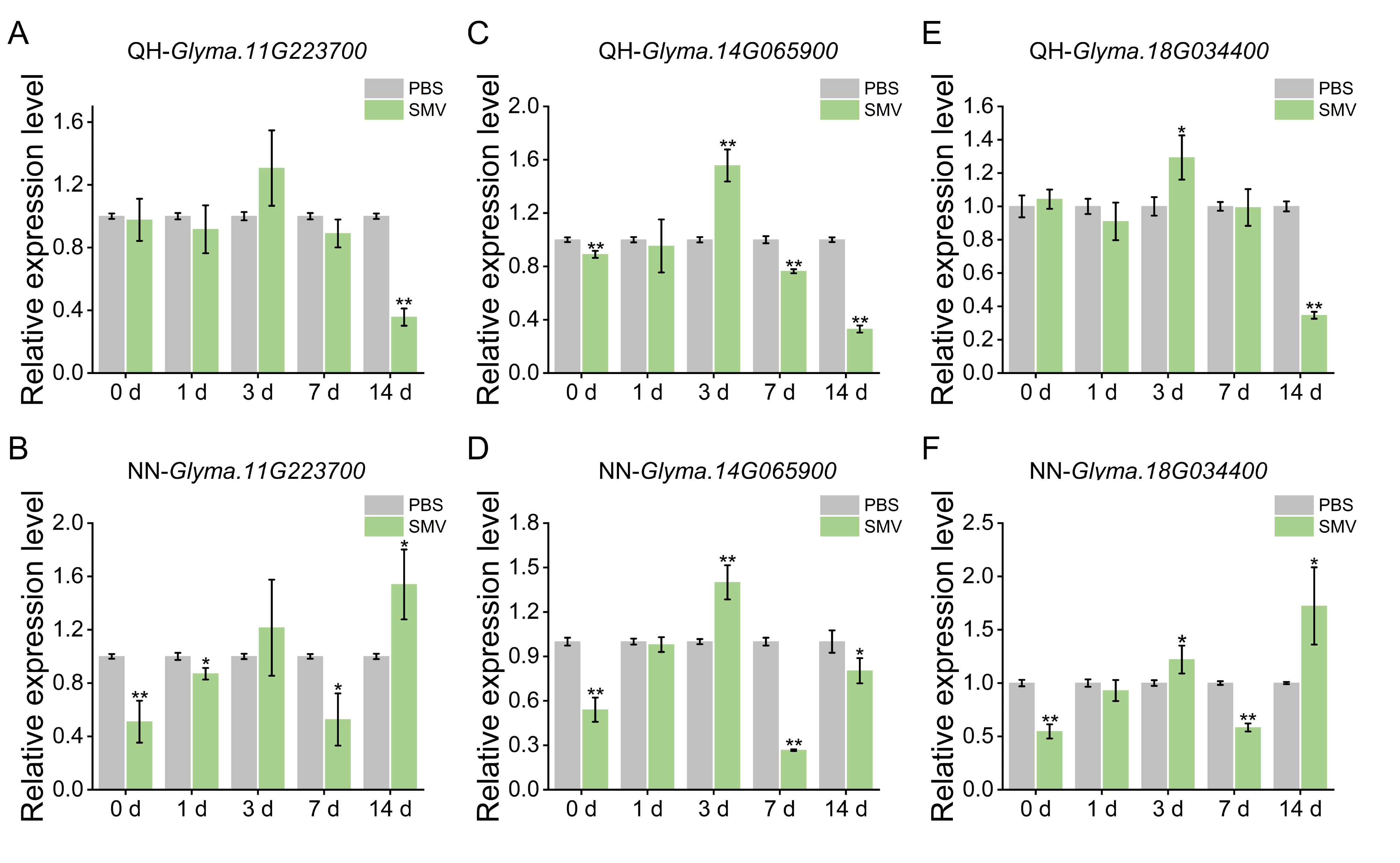

Supplement: Supplementary file 1 [file plants-15-01020-s001.zip › Figures/Figure S3-revision.tif]
